# Supplementary material for: Conserved visual capacity of rats under red light
Source: eLife. 2021 Jul 20;10:e66429. doi: 10.7554/eLife.66429 (PMC8360654; doi:10.7554/eLife.66429)
Supplement: Supplementary file 1. — Measured intensity of each LED in mW/cm2 and total irradiated power (in mW), as measured through our spectrometer (Supplementary table 1) and Additional LED emission characteristics (Supplementary table 2). [file elife-66429-supp1.docx]

# Supplementary File 1

| **1. LED type** | **2. Intensity (mW/cm^2^)** | **3. Total measured irradiated power (mW)** | **4. Applied forward current (mA)** | ***5. Total radiated power and current rating from manufacturer datasheet** | ***6. Expected radiated power at test current (mw)** |
| --- | --- | --- | --- | --- | --- |
| White LED | 1417.11 | 169.3 | 15 | N/A | N/A |
| RED 626 nm | 19.65 | 2.4 | 10 | 120 mW at 240 mA | 5 |
| RED 652 nm | 55.63 | 6.6 | 10 | 180 mW at 240 mA | 7.5 |
| RED 729 nm | 9.59 | 1.2 | 8 | 180 mW at 240 mA | 6 |
| IR 854 nm | 299.55 | 35.8 | 20 | 1500 mW at 800 mA | 37.5 |
| IR 930 nm | 77.11 | 9.1 | 10 | 550 mW at 600 mA | 9.2 |

**Supplementary table 1.** Measured intensity of each LED in mW/cm^2^ and total irradiated power (in mW), as measured through our spectrometer (fiber+cosine corrector with a diameter of 3900 µm) from direct measurement of the LEDs, together with the forward current with which the LEDs were supplied and the power rating from the manufacturer datasheet.

*The LED datasheets report total radiated power when the LED is powered up at the rated test currents (table column 5). Since actual irradiation scales roughly linearly with the applied current, we can compute the expected radiated power at the current used in our setup according to the datasheet (table column 6).

| **LED type** | **Total power density**  **(µW/cm^2^)** | **Photon flux density at LED (photons/μm^2^/s)** | **Power density received by rat’s eye (µW/cm^2^)** | **Photon flux density at rat’s eye (photons/μm^2^/s)** | **Photon flux density at photoreceptor (photons/μm^2^/s)** | **Photon flux at photoreceptor (photons/s)** |
| --- | --- | --- | --- | --- | --- | --- |
| White LED | 1.14e+06 | 3.04e+19 | 2.28e+0 | 6.08e+13 | 1.19e+05 | 4.78e+05 |
| RED 626 nm | 1.12e+04 | 3.52e+17 | 2.25e-02 | 7.04e+11 | 1.38e+03 | 5.53e+03 |
| RED 652 nm | 3.36e+04 | 2.20e+12 | 6.72e-02 | 2.20e+12 | 4.31e+03 | 1.73e+04 |
| RED 729 nm | 6.78e+03 | 2.48e+17 | 1.36e-02 | 4.96e+11 | 9.75e+02 | 3.90e+03 |
| IR 854 nm | 2.34e+05 | 9.99e+18 | 4.68e-01 | 2.00e+13 | 3.92e+04 | 1.57e+05 |
| IR 930 nm | 7.59e+04 | 3.56e+18 | 1.52e-01 | 7.12e+12 | 1.40e+04 | 5.59e+04 |

**Supplementary table 2. Additional LED emission characteristics.**
