# Supplementary material for: Conserved visual capacity of rats under red light
Source: eLife. 2021 Jul 20;10:e66429. doi: 10.7554/eLife.66429 (PMC8360654; doi:10.7554/eLife.66429)
Supplement: Transparent reporting form [file elife-66429-transrepform.pdf]

eLIFE
1st Floor
24 Hills Road
Cambridge CB2 1JP, UK
P 01223 855340
W elifesciences.org
T @elife

eLife's transparent reporting form

We encourage authors to provide detailed information *within their submission* to facilitate the interpretation and replication of experiments. Authors can upload supporting documentation to indicate the use of appropriate reporting guidelines for health-related research (see EQUATOR Network), life science research (see the BioSharing Information Resource), or the ARRIVE guidelines for reporting work involving animal research. Where applicable, authors should refer to any relevant reporting standards documents in this form.

If you have any questions, please consult our Journal Policies and/or contact us: editorial@elifesciences.org.

Sample-size estimation

- You should state whether an appropriate sample size was computed when the study was being designed
- You should state the statistical method of sample size computation and any required assumptions
- If no explicit power analysis was used, you should describe how you decided what sample (replicate) size (number) to use

Please outline where this information can be found within the submission (e.g., sections or figure legends), or explain why this information doesn't apply to your submission:

In this study we performed behavioral measurements of rats' visual discrimination capacity under different lighting conditions. We trained rats using established behavioral training protocols detailed in Nikbakht et al., 2018. ALL rats learned the task and achieved stable performance with 70%-90% accuracy.
To quantify rats' performance, we used a cumulative Gaussian function to fit psychometric curves to the choice data of each rat. To evaluate performance in each condition with 95% confidence (z-score for a 95% confidence level is 1.96) and a margin of error (ε) of 10% and a population proportion ($\hat{p}$) of 0.9 we will need:

$$n = \frac{z^2 \times \hat{p}(1-\hat{p})}{\varepsilon^2} \rightarrow n = \frac{1.96^2 \times 0.9(1-0.9)}{0.1^2} \cong 35$$

At least 35 trials per experimental condition. Our dataset consisted of at least ~300 trials in any given experimental condition (each stimulus orientation in each lighting condition). The statistical power and very low sampling noise of our results thus comes from the thousands of trials performed by each rat, and not from the sample size in the sense of the number of animals tested. We tested Four male Long–Evans rats; they were randomly selected to be part of the experiment and none were excluded from the study as they all exhibited robust and unbiased performance a clear indication as to whether the results obtained are reproducible across different individual animals.
The details of the behavioral methods and statistical analyses can be found in the Methods section of the paper.

eLife Sciences Publications, Ltd is a limited liability non-profit non-stock corporation incorporated in the State of Delaware, USA, with company number 5030732, and is registered in the UK with company number FC030576 and branch number BR015634 at the address 1st Floor, 24 Hills Road, Cambridge CB2 1JP | August 2014

Replicates

- You should report how often each experiment was performed
- You should include a definition of biological versus technical replication
- The data obtained should be provided and sufficient information should be provided to indicate the number of independent biological and/or technical replicates
- If you encountered any outliers, you should describe how these were handled
- Criteria for exclusion/inclusion of data should be clearly stated
- High-throughput sequence data should be uploaded before submission, with a private link for reviewers provided (these are available from both GEO and ArrayExpress)

Please outline where this information can be found within the submission (e.g., sections or figure legends), or explain why this information doesn't apply to your submission:

Our Material & Methods and our Results sections contain detailed information answering all these questions about replication. In our study rats were trained for 4–6 weeks for 5 times a week and generated between 300-500 trials per day. All animals were included in the study.

Statistical reporting

- Statistical analysis methods should be described and justified
- Raw data should be presented in figures whenever informative to do so (typically when N per group is less than 10)
- For each experiment, you should identify the statistical tests used, exact values of N, definitions of center, methods of multiple test correction, and dispersion and precision measures (e.g., mean, median, SD, SEM, confidence intervals; and, for the major substantive results, a measure of effect size (e.g., Pearson's r, Cohen's d)
- Report exact p-values wherever possible alongside the summary statistics and 95% confidence intervals. These should be reported for all key questions and not only when the p-value is less than 0.05.

Please outline where this information can be found within the submission (e.g., sections or figure legends), or explain why this information doesn't apply to your submission:

The statistical analysis of our behavioral data is described in detail in the section "Quantification and Statistical Analysis" of the Methods section and the results of the analysis are presented in figures 1 and 2 and explained in the Results section.

(For large datasets, or papers with a very large number of statistical tests, you may upload a single table file with tests, Ns, etc., with reference to sections in the manuscript.)

Group allocation

- Indicate how samples were allocated into experimental groups (in the case of clinical studies, please specify allocation to treatment method); if randomization was used, please also state if restricted randomization was applied
- Indicate if masking was used during group allocation, data collection and/or data analysis

Please outline where this information can be found within the submission (e.g., sections or figure legends), or explain why this information doesn't apply to your submission:

eLife Sciences Publications, Ltd is a limited liability non-profit non-stock corporation incorporated in the State of Delaware, USA, with company number 5030732, and is registered in the UK with company number FC030576 and branch number BR015634 at the address 1st Floor, 24 Hills Road, Cambridge CB2 1JP | August 2014

Rats were randomly selected to be part of the experiment and none were excluded from the study. The collection and the analysis of the behavioral data was performed in an automated manner, LabVIEW (National Instruments, Austin, TX), an AVR32 board (National Instruments) and multiple Arduino Shields (National Instruments).

Additional data files ("source data")

- We encourage you to upload relevant additional data files, such as numerical data that are represented as a graph in a figure, or as a summary table
- Where provided, these should be in the most useful format, and they can be uploaded as "Source data" files linked to a main figure or table
- Include model definition files including the full list of parameters used
- Include code used for data analysis (e.g., R, MatLab)
- Avoid stating that data files are "available upon request"

Please indicate the figures or tables for which source data files have been provided:

We will upload all the source data and analysis source code to a public depository such as Github as soon as our paper is accepted for publication.
